# Dynamics of Monoterpene Formation in Spike Lavender Plants

**DOI:** 10.3390/metabo7040065

**Published:** 2017-12-19

**Authors:** Isabel Mendoza-Poudereux, Erika Kutzner, Claudia Huber, Juan Segura, Isabel Arrillaga, Wolfgang Eisenreich

**Affiliations:** 1 Departamento de Biología Vegetal, ISIC/ERI de Biotecnología y Biomedicina BIOTECMED, Universidad de Valencia, Av. Vicent Andrés Estellés s/n, 46100 Burjasot, Valencia, Spain; juan.segura@uv.es (J.S.); isabel.arrillaga@uv.es (I.A.); 2 Lehrstuhl für Biochemie, Technische Universität München, Lichtenbergstraße 4, 85748 Garching, Germany; erika.lackermeier@mytum.de (E.K.); claudia.huber@mytum.de (C.H.); wolfgang.eisenreich@mytum.de (W.E.)

**Keywords:** essential oils, isotopologue profiling, lamiaceae, *Lavandula latifolia*, spike lavender, terpenoid biosynthesis, mevalonate, CO_2_

## Abstract

The metabolic cross-talk between the mevalonate (MVA) and the methylerythritol phosphate (MEP) pathways was analyzed in spike lavender (*Lavandula latifolia* Med) on the basis of ^13^CO_2_-labelling experiments using wildtype and transgenic plants overexpressing the 3-hydroxy-3-methylglutaryl CoA reductase (HMGR), the first and key enzyme of the MVA pathway. The plants were labelled in the presence of ^13^CO_2_ in a gas chamber for controlled pulse and chase periods of time. GC/MS and NMR analysis of 1,8-cineole and camphor, the major monoterpenes present in their essential oil, indicated that the C5-precursors, isopentenyl diphosphate (IPP) and dimethylallyl diphosphate (DMAPP) of both monoterpenes are predominantly biosynthesized via the MEP pathway. Surprisingly, overexpression of HMGR did not have significant impact upon the crosstalk between the MVA and MEP pathways indicating that the MEP route is the preferred pathway for the synthesis of C5 monoterpene precursors in spike lavender.

## 1. Introduction

Terpenes are the largest and most diverse family of natural products. Within these compounds both primary metabolites (hormones, carotenoids, chlorophylls and sterols), necessary for plant growth and survival, and secondary metabolites, that are not directly involved in growth and/or development, can be found [1,2,3,4,5]. The latter compounds have also considerable commercial importance due to their uses in food, perfume, cosmetic and pharmaceutical industries [6,7,8,9]. 

**Figure 1:**
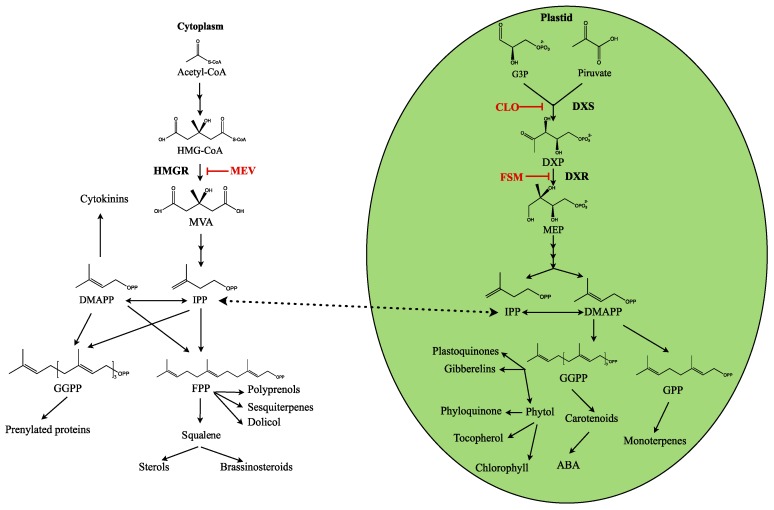
General scheme of the terpene synthesis pathways in plants and their inhibitors. ABA: abscisic acid. CLO: clomazone. DMAPP: isomer dimethylallyl diphosphte. DXP: 1-deoxy-d-xylulose 5-phosphate. DXR: DXP reductoisomerase. DXS: DXP synthase. FPP: farnesyl diphosphate. FSM: fosmidomycin. GGPP: geranylgeranyl diphosphate. G3P: d-glyceraldehyde-3 phosphate. HMG-CoA: 3-hydroxy-3-methylglutaryl-coenzyme A. HMGR: HMG-CoA reductase. IPP: isopentenyl diphosphate. MEP: methyl-d-erytritol-4-phosphate. MEV: mevinoline. MVA: mevalonate [10,11]

All terpenes originate from the universal C5 precursor IPP and its isomer DMAPP. In plants, both compounds are formed through two pathways [10,11]: the cytosolic MVA pathway, which comprises several enzymatic reactions from acetyl coenzymeA condensation through mevalonate iphosphate decarboxylation, and the plastidial MEP pathway starting from the reaction between pyruvate and glyceraldehyde-3-phosphate (Figure 1). 

The MVA pathway is primarily regulated at the level of the 3-hydroxy-3-methylglutaryl-coenzyme A (HMG-CoA) reductase (HMGR) [1,12], and it is known that HMGR activity regulates the metabolic flux through the MVA pathway and the eventual production of the isoprenoid end-products [13,14]. The scenario seems to be more complex in the case of the MEP pathway. In fact, the MEP pathway can be regulated by several enzymes, including 1-deoxy-d-xylulose 5-phosphate (DXP) synthase (DXS), DXP reductoisomerase (DXR) [15] and hydroxymethyl-butenyl 4-diphosphate (HMBPP) reductase (HDR) [13,15,16]. 

Generally, it is assumed that the MVA pathway is responsible for providing precursors for the synthesis of sesquiterpenes and triterpenes, while the MEP pathway donates precursors for monoterpenes, diterpenes and tetraterpenes [17]. Although both pathways are thought to operate independently under normal conditions, interactions between them have been repeatedly reported [18]. Thus, experiments with labelled products and/or treatments with specific inhibitors (mevinoline and fosmidomycin that respectively block the MVA and MEP pathways) illustrate that the compartmental division between the two pathways is not complete since common metabolites from both pathways can be exchanged, in both directions, through the plastidial membrane [11,19,20]. Therefore, the relative contribution of the MVA and MEP pathways for the biosynthesis of plant terpenes and the factors controlling this distribution especially under physiological conditions of growing plants remain uncertain for each plant species. It is still assumed, however, that monoterpenes are primarily synthesized in the plastids via the MEP pathway-derived IPP and DMAPP [21].

**Figure 2:**
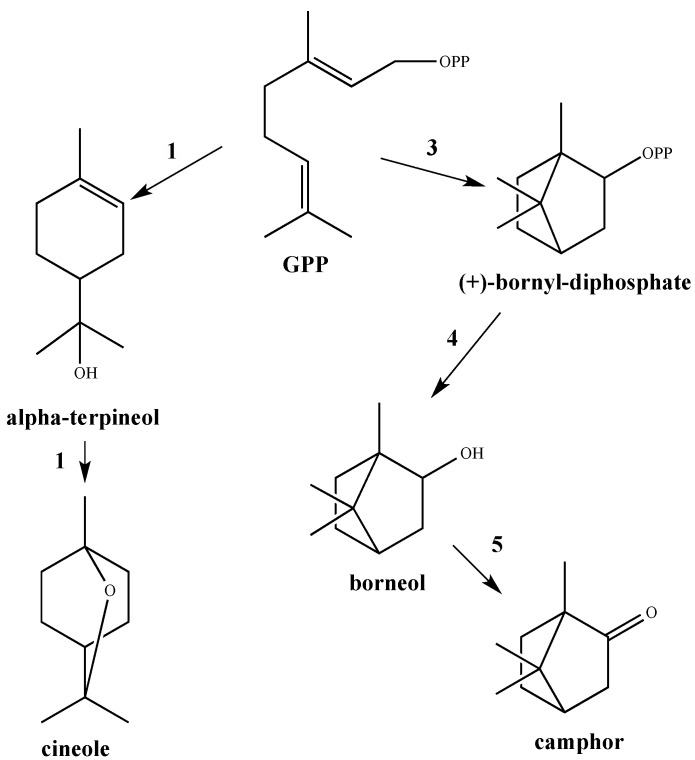
Scheme for the synthesis of the monoterpenes cineole and camphor. GPP, geranyl diphosphate; 1, cineole synthase; 2, bornyl diphosphate synthase; 3, bornyl diphosphate diphosphatase; 4, borneol dehydrogenase. Adapted from [22,23,24,25].

Spike lavender (*Lavandula latifolia* Medicus) is an aromatic shrub of high economic importance due to its essential oil, composed mainly of monoterpenes [22]. The main constituents in spike lavender oil from flowers are the monoterpenes linalool, cineole and camphor, while the main constituents in the oil from leaves are cineole and camphor [23]. All monoterpenes are derived from geranyl diphosphate molecule (GPP). In the case of camphor, GPP is transformed via cyclisation of linaloyl pyrophosphate into bornyl pyrophosphate, followed by a hydrolysis reaction that produces borneol, which finally is oxidized to camphor. In the case of cineole, the GPP intermediate is first converted into α-terpineol by the α-terpineol synthase and this monoterpene is converted into cineole by the cineole synthase [24] (Figure 2).

Due to its high economic interest, the composition and quality of spike lavender oil has been widely studied including chemical composition of essential oil from some Spanish wild populations [26,27,28,29]. All these studies revealed a great intraspecific variability in the chemical composition of oils that can be attributed to several variation sources: genotypic, climatic, geographical and/or seasonal [28,29,30,31,32,33].

Modifications of yield and composition of the spike lavender essential oil by genetic engineering may have important scientific and commercial applications. Until now, three genes involved in the first steps of the synthesis of terpenes have been overexpressed in spike lavender, the *Arabidopsis* HMG1, DXS and DXR genes, encoding the respective HMGR1S, DXS and DXR enzymes. As expected, the overexpression of the DXS gene produced a significant increase of the end-product monoterpenes derived from an elevated supply of precursors of the MEP isoprenoid pathway without affecting the chlorophyll and carotenoid content [34]. Surprisingly, the overexpression of the DXR enzyme did not lead to an increase of monoterpenes or photosynthetic pigments [35]. In contrast, the overexpression of the HMG1 gene from *Arabidopsis*, led to an increased yield of essential oil since transgenic T0 plants accumulated significantly more essential oil constituents as compared to controls (up to 2.1- and 1.8-fold in leaves and flowers, respectively). These transgenic plants also increased its amount of the end-product sterols, β-sitosterol and stigmasterol (average differences of 1.8- and 1.9-fold, respectively), but did not accumulate more carotenoids or chlorophylls as compared to controls. The potential of the mevalonate pathway for enhanced isoprenoid production by upregulation of HMGR and other enzymes of the pathway as well as its interaction with the MEP pathway have been updated recently [36]. The increased levels of essential oil and sterols observed in the transgenic T0 plants were maintained in the progeny that inherited the HMG1 transgene. Therefore, genetic manipulation of the MVA pathway also increases essential oil yield in spike lavender, suggesting a contribution for this cytosolic pathway to monoterpene and sesquiterpene biosynthesis in leaves and flowers of the species [37]. 

The incorporation of ^13^C- or ^14^C-labelled tracer compounds into the current metabolism is one of the most powerful tools to determine the metabolites biosynthetic pathways. To study primary and secondary metabolism in plants, general precursors (e.g., glucose) or more specific intermediates can be used as tracers and supplied to plant tissues maintained either in vitro (e.g., plant cell cultures, stem or leave cultures, seedlings) [38,39] or ex vitro [39]. By studying the presence and distribution of the labelled atoms in the final products, it is possible to reconstruct the metabolic pathways followed [39].

The use of ^13^CO_2_ as a tracer is a powerful tool, since this strategy represents the possibility of studying metabolic pathways in plants by mimicking, as much as possible, their physiological growth conditions [40,41,42]. The labelling period (pulse) is followed by a chase period in which the plants grow under normal conditions and natural ^13^CO_2_ abundance for several hours/days. During that time, unlabeled products and intermediates are produced, that also are used in the production of the final metabolites. Therefore, complex patterns of [^12^C]- and [^13^C]-isotopologues are expected to be found in the final natural products. The analysis of complex isotopologue mixtures in ^13^CO_2_-labelled natural products became possible recently, because of the remarkable improvement made in NMR and mass spectrometry techniques [40,42,43,44,45,46]. ^13^CO_2_ feeding has also been used to measure plastidic DMAPP concentrations and to quantify the *de novo* production of volatile and nonvolatile isoprenoids in some species [47]. In this paper, we describe the ^13^CO_2_ labelling conditions necessary to study the incorporation of ^13^CO_2_ into cineole and camphor in wild type (WT) and HMGR5 transgenic spike lavender plants. The results are aimed to clarify the possible contribution of the MVA pathway to the biosynthesis of both monoterpenes in the species. 

## 2. Results

### 2.1. GC/MS Analysis

GC/MS analysis of chloroform extracts from WT plants allowed the detection of most of the components of the spike lavender essential oil [48,49] including monoterpenes, sesquiterpenes and, at longer retention times, over 21 min, coumarin, as previously reported [37,49] (data not shown). 

Over 50 samples were used in order to adjust the extraction, labelling and GC/MS measuring conditions (Table S1). 

**Figure 3:**
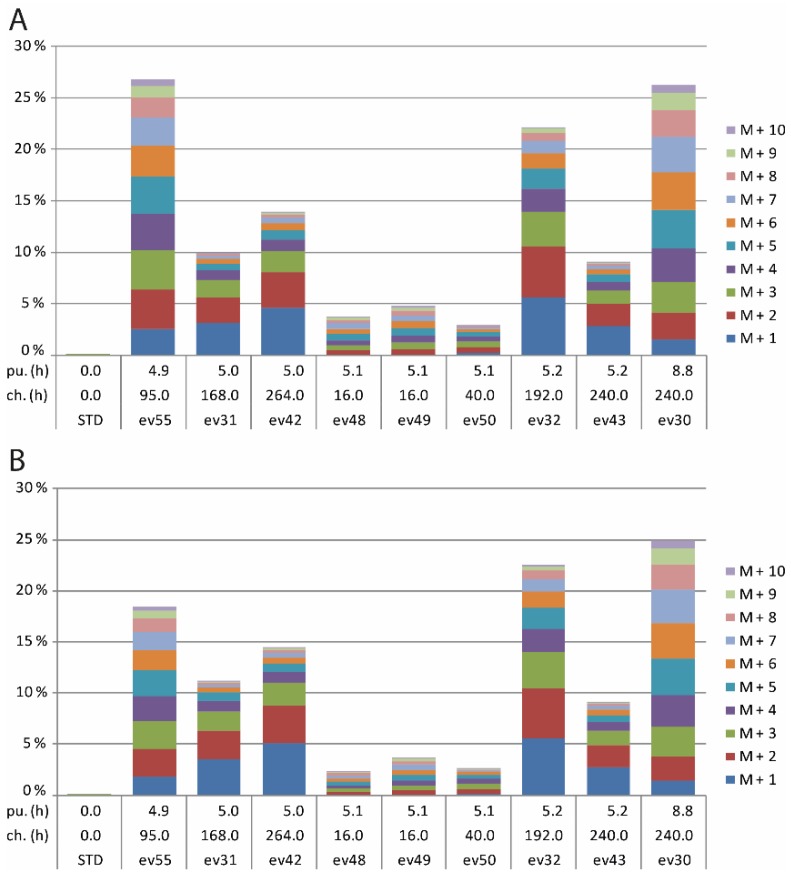
(**A**) Isotopologue excess values and distribution of isotopomers of cineole for selected samples in ^13^CO_2_ feeding experiments. All experiments are sort according to their chase time in ascending order. pu. (h): pulse time in hours. ch. (h): chase time in hours. STD: pure cineole sample. (**B**) Isotopologue excess values and distribution of isotopomers of camphor for selected samples in ^13^CO_2_ feeding experiments. All experiments are sort according to their chase time in ascending order. pu. (h): pulse time in hours. ch. (h): chase time in hours. STD: pure camphor sample.

Total excess of ^13^C abundance and the relative contributions of isotopomers (containing 1 or more ^13^C-atoms, indicated by M+1, M+2, …, M+10, respectively) in the ^13^C-enriched fraction of camphor and 1,8-cineole of selected samples are depicted in Figure 3A,B.

More specifically, the labelling profiles shown in Figures S1 and S2 indicated that a pulse period below five hours did not lead to significant ^13^C enrichments in both camphor and 1,8-cineol. However, ^13^CO_2_ pulses of five hours and more resulted in ^13^C-enrichments of >2% (Figure 3A,B). Some of the samples showed ^13^C enrichments of more than 10% (up to 25% in some cineole and camphor samples (Figure 3). Even experiments with a given pulse period (i.e., the said 5 h) showed considerable differences of labelling profiles that can be explained, in part, on the basis of different chase periods. Generally, it appeared that the ^13^C enrichments increase with higher chase periods (Figures S1 and S2).

To interpret the data in more detail, samples 48, 49, 50, 55, 31, 32, 43, 42 and 30, all of them with isotopologue excess values over 2.5% were chosen (Figure 3 and Table 1). Values for the isotopomers M+2 and M+3 and their ratios for both camphor and cineole are presented in Table 1. In all of these samples, the M+1 to M+3 fraction ranged from 25–73% of total labelled excess isotopologues.

### 2.2. NMR Data

**Figure 4:**
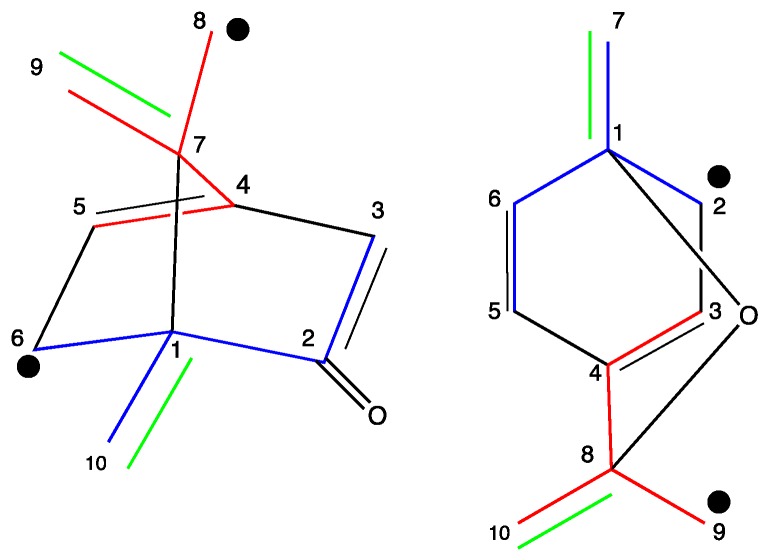
Numbering and biosynthetic origin of carbon atoms from camphor and 1,8-cineole synthesized via the MEP pathway. Carbon atoms derived from: DMAPP (red), IPP (blue), Pyruvate (Green), G3P (Black).

The positional distribution of the labels in cineole and camphor (Figure 4) were determined by ^13^C NMR spectroscopy (Table 2 and Table 3). Notably, NMR signals for camphor and 1,8-cineole could be detected with the crude chloroform extracts of the leaves by comparison with reference data and on the basis of two-dimensional NMR experiments (Table 2, Table 3 and Table 4). The overall ^13^C-enrichments of carbon atoms were similar and in the range of 2–10% for most of the samples. Unfortunately, the ^13^CO_2_ labelling experiments afforded similar, if not identical, ^13^C-enrichments for the various carbon positions in the monoterpenes, as exemplified for cineole (Table S2). However, the generation of multiply ^13^C-labelled isotopologues was dependent on the trunk biosynthetic pathways, as outlined below.

**Figure 5:**
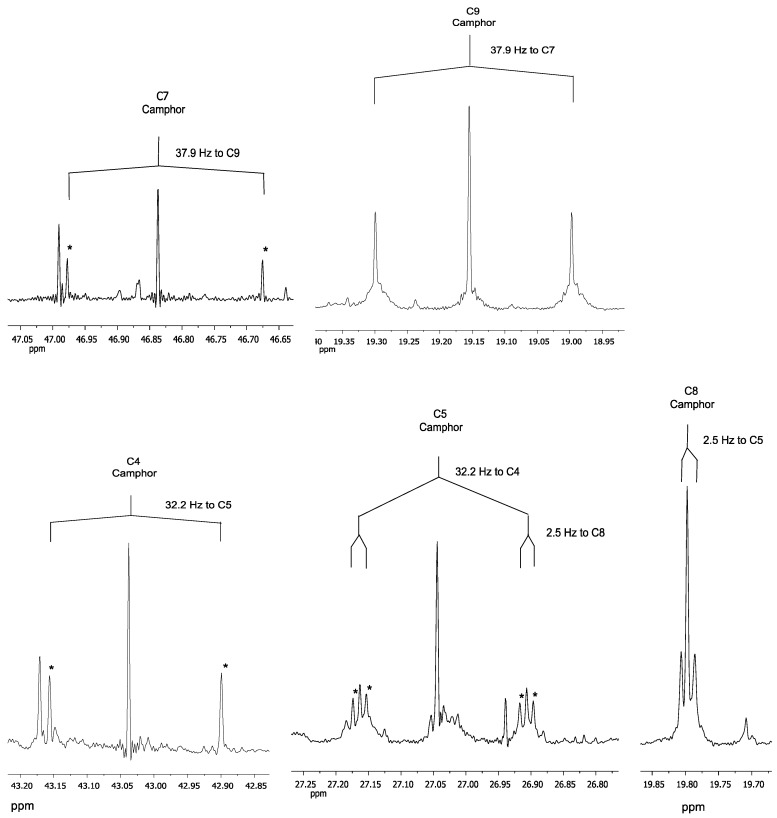
^13^C NMR camphor signals, chloroform-d leaf essential oil extract from spike lavender after incorporation of ^13^CO_2_ (pulse 5.2 h, chase 192 h). * indicate the satellite signals of the carbon atoms under study.

**Figure 6:**
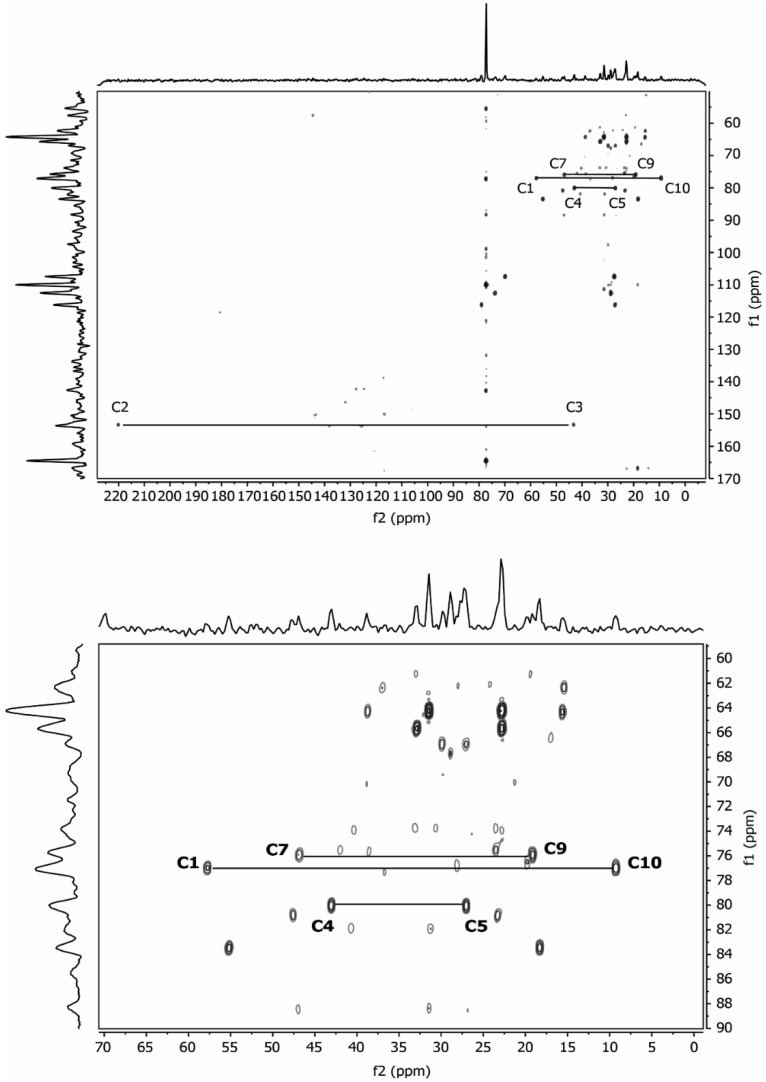
INADEQUATE ^13^C NMR camphor signals; chloroform-d leaf essential oil extract from spike lavender after incorporation of ^13^CO_2_ (pulse 5.2 h, chase 192 h. (NMR parameter: pulse program: inadqf, TD1: 300, NS: 128, J (CC): 50 Hz, D1: 2 s), measurement time: 22 h.

**Figure 7:**
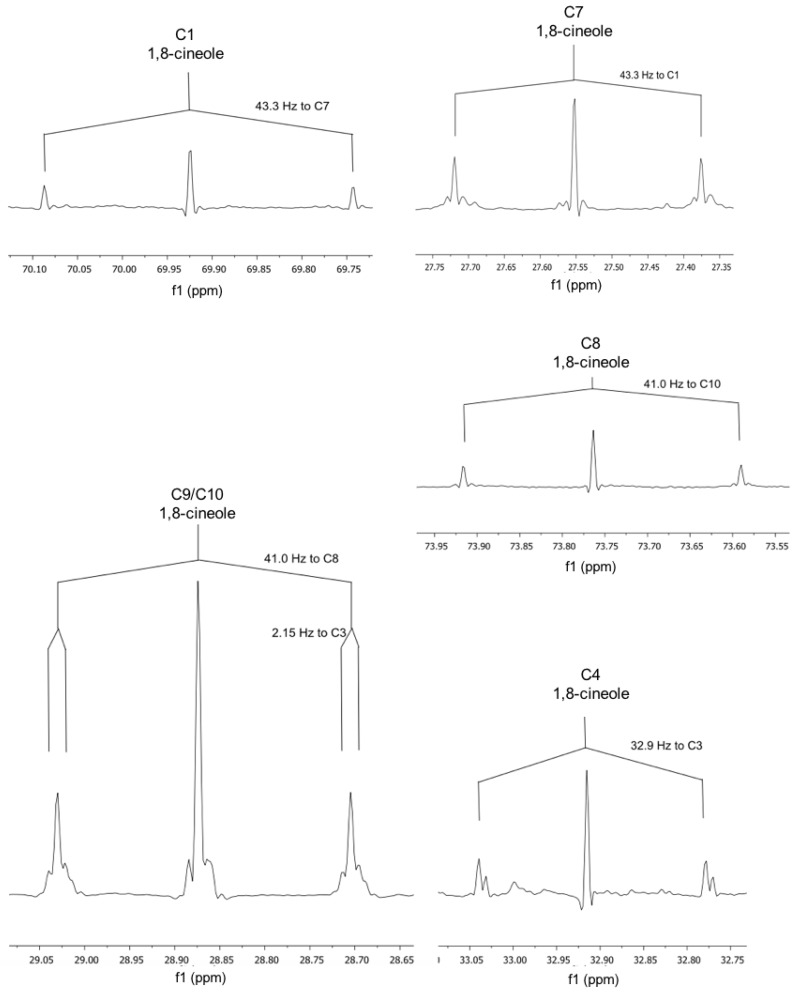
^13^C NMR 1,8-cineole signals; chloroform-d leaf essential oil extract from spike lavender after incorporation of ^13^CO_2_ (pulse 5.2 h, chase 192 h).

Both monoterpenes displayed ^13^C-coupled satellites due to the presence of multiply ^13^C-labelled isotopologues. On the basis of the specific coupling constants and the detection of specific correlation patterns in INADEQUATE and 1,1-ADEQUATE experiments (Figure 5, Figure 6 and Figure 7), it can be concluded that the isotopologue profiles of both compounds were highly specific. As shown in Table 2, Table 3 and Table 4 and Figure 5, Figure 6 and Figure 7, we assigned 10 and 7 ^13^C_2_-isotopologues in camphor and cineole, respectively. Moreover, the detection of long-range ^13^C-couplings lead to the assignment of ^13^C-isotopologues in camphor and cineole, as well (Table 2, Table 3 and Table 4 and Figure 5, Figure 6 and Figure 7).

In the case of camphor, the ten C atoms from the monoterpene can be undoubtedly distinguished in the spectrum (Table 4). Coupling constants of satellite pairs C1–C10, C9–C7, C8–C5, C3–C2 and C4–C8 can be clearly attributed (see Figure 5 and Figure 6 and Table 4). The signal for carbon atom C5 shows a satellite pair caused by coupling to C4 (32.2 Hz) and an additional coupling to C8 (2.5 Hz), reflected by a splitting of the satellite pair. Only the long-range coupling of the carbonyl-C2 to C6 cannot be resolved in detail. These results were confirmed by the INADEQUATE experiment, which is shown in Figure 6. Clearly, Figure 6 shows all the couplings already described in Figure 5. The long-range coupling of the carbonyl-C2 to C6 cannot be resolved in the INADEQUATE spectrum as well.

Regarding cineole, the 10 C atoms were also determined; as expected, satellite pairs C3–C5, C9–C10, and C2–C6 are indistinguishable from each other because of the circular symmetric structure of the monoterpene. Coupling constants of satellite pairs caused by C3–C4, C7–C1, C10–C8, C9–C4, and C10/C9 can be clearly attributed (see Figure 7). A long-range coupling caused by C9 to C3 is also detected. It is possible that the signal of C3/C5 overlays with a α-pinene signal. 

All camphor coupling constants are in concordance with the addition of a labelled IPP derived from G3P into the molecule through the MEP terpene synthesis pathway and not through IPP produced by the MVA pathway. The analysis of cineole provides enough evidence to also support the hypothesis that the MEP pathway is responsible for the biosynthesis of 1,8-cineole due to the C9–C3 long-range coupling detected; this triple is only expected for a moiety where G3P serves as a precursor.

### 2.3. ^13^CO_2_ Labelling in Transgenic HMGR5 Plants

WT and HMGR5 plants were labelled with ^13^CO_2_ for 5 h and harvested at different chase times. The terpenes were extracted and analyzed by GC/MS as previously described. 

Transgenic HMGR5 accumulated more monoterpenes than the WT (Table 5). The percentages of the five main components of the leaf essential oil (the monoterpenes cineole, camphor, α-pinene, β-pinene and limonene) were considered in relation to the 15 main peaks of each sample and are presented in Table 5. These five molecules accounted for almost 88% of the 15 peaks in the HMGR5 plants and 78% for the WT plants, revealing that apparently the HMGR5 plants had a higher proportion of monoterpenes in their essential oil. Coumarine was also detected in most samples (Table S3), elevating the total percentage of identified compounds to over 94% in the case of HMGR5 plants and 88% for WT plants. The main compounds in all samples were camphor and 1,8-cineole that accounted for a total of 79% in the case of HMGR5 plants and less than 67% in the case of WT plants. These results are in accordance with those previously described by our group, with HMGR overexpressing plants that accumulated significantly more essential oil constituents, mainly monoterpenes and sesquiterpenes, as compared to controls. Enhanced expression of HMGR1S also increased the amount of the end-product sterols, β-sitosterol and stigmasterol, but did not affect the accumulation of carotenoids or chlorophylls [37]. 

Although all plants were labelled for a pulse time of five hours, low excess ^13^C incorporation was achieved, between 0.01% and 0.83% (Table S4). This might be due to a poor watering of the plants that may induce the stomata closing and a photosynthetic reduction. Nevertheless, some of the obtained results can be discussed.

**Figure 8:**
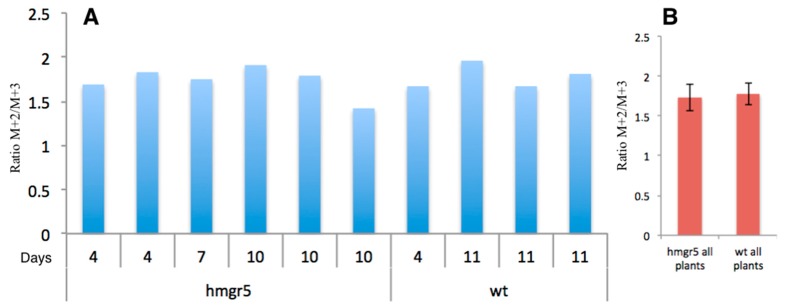
^13^C enrichment in 1,8-cineole. Excess values of M+2 and M+3 isotopomeres obtained from GC/MS analysis from spike lavender leaf essential oil (chloroform-d extracts) labelled with ^13^CO_2_ for 5 h. (**A**) Ratios of M+2 and M+3 calculated for 6 HMGR5 transgenic plants and 4 wild type (WT) plants. (**B**) Mean values of the M+2/M+3 ratios for all HMGR5 plants and all WT plants.

**Figure 9:**
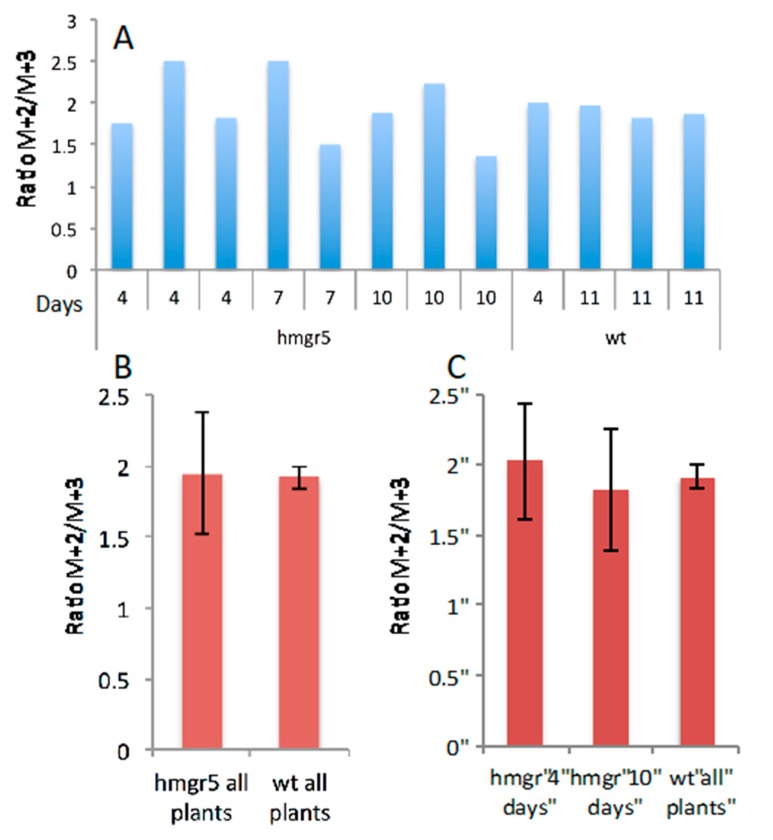
^1^^3^C enrichment in camphor. Excess values of M+2 and M+3 isotopomeres obtained from GC/MS analysis from spike lavender leaf essential oil (chloroform-d extracts) labelled with ^13^CO_2_ for 5 h. (**A**) Ratios of M+2 and M+3 calculated for 8 HMGR5 transgenic plants and 4 wild type (WT) plants. (**B**) Mean ± SD values of the M+2/M+3 ratios for all HMGR5 plants and all WT plants. (**C**) Mean ± SD values of the M+2/M+3 ratios for all HMGR5 plants harvested 4 and 10 days after labelling and all WT plants harvested 11 days after labelling.

The M+2 and M+3 ratios for 1,8-cineole and camphor are displayed in Figure 8A and Figure 9A and Table 6 and Table 7, respectively. These ratios were determined for eight transgenic plants and for four wild type plants. Three HMGR5 plants were harvested 96 h after the pulse time, two after 168 h and three after 240 h. The WT plants were harvested after 96 h (one plant) and 264 h (three plants). Only harvested plants showing an excess of ^13^C incorporation to 1,8-cineole are included in Figure 8A. Mean results for cineole revealed that HMGR5 and WT plants have very similar M+2/M+3 ratios (1.7 ± 0.2 and 1.8 ± 0.1, respectively) (Figure 8B). Similar results were observed in the M+2/M+3 ratios for camphor in WT plants (mean ratio value of 1.9 ± 0.1; Figure 9B). In contrast, HMGR5 plants showed a high deviation in the M+2/M+3 ratio within the same chase group (mean value for the ratios were 2.0 ± 0.4 and 1.8 ± 0.4 for 4 and 10 days chase periods, respectively; Figure 9C). 

## 3. Discussion

### 3.1. GC/MS Analysis

All samples analyzed showed a quite stable M+2/M+3 ratio of 1.2 ± 0.3 for camphor and 1.2 ± 0.3 for cineole. Also, the M+2/(M+2 + M+3) ratio was very stable (0.5 ± 0.1 for camphor and 0.5 ± 0.1 for cineole). If both monoterpenes are produced exclusively through the MEP pathway the expected values for their ratios would be 1 and 0.5 respectively. If the MVA pathway is involved, the ratios would rise above those numbers, because of the presence of more M+2 coming from IPP from the cytosolic space. The observed values are therefore in the range of those expected for monoterpene biosynthesis mainly via the MEP pathway in *Lavandula latifolia* under this culture conditions. 

### 3.2. NMR Data

NMR data show that in *Lavandula latifolia* precursors for the biosynthesis of camphor and 1,8-cineole are predominantly provided via the MEP pathway. This conclusion is not only highlighted by the NMR results from the sample 32 (Table 4 and Figure 7, Figure 8 and Figure 9), but also by the M+2 and M+3 values and their ratios (Table 1). This metabolic behavior has been described in most plants, where MEP-derived precursors contributed, almost exclusively, to the synthesis of monoterpenes, diterpenes and carotenoids [50]. Note, however, that in glandular trichomes of peppermint, another essential oil-producing species like spike lavender, it has been determined that the MVA pathway is blocked at HMGR [51], thus, in this species, the MEP pathway seems to be the exclusive source of precursors for monoterpene biosynthesis [52]. 

### 3.3. ^13^CO_2_ Labelling in Transgenic HMGR5 Plants

Metabolic engineering studies in spike lavender demonstrated that MEP and MVA pathways may be implicated in the monoterpene biosynthesis. Nevertheless, essential oil yield was always higher in those transgenic spike lavender plants overexpressing the DXS gene. Previous work using specific inhibitors of both pathways and feeding experiments with [U-^13^C6]-glucose, suggested a minor contribution of the MVA pathway that was increased in transgenic (HMGR5) spike lavender plants [37]. Results presented here using labelled ^13^CO_2_ confirmed that in WT and transgenic HMGR spike lavender plants, the biosynthesis of camphor and cineole happen in a very robust way through the MEP pathway according to the results achieved with NMR and GC/MS. Still, outcomes with HMGR overexpressing spike lavender plants cast doubt about the fact that the MVA pathway might be involved, even in a small share, on the production of monoterpenes because of the higher content of essential oil in those plants. It is well known that the MVA and MEP pathways are not totally independent as there is some crosstalk between them through common intermediates [11,39,53]. Experiments with labelled intermediates [2-^13^C_1_]mevalonolactone or [U-^13^C_6_]glucose in *Catharanthus roseus* using NMR techniques showed that crosstalk between the MVA and MEP pathways cannot be explained in detail by a simple two compartment model and requires an additional in depth study of complex regulatory mechanisms [19]. 

For some time, ^13^CO_2_ has been used several times as a tool for the analysis of quantitative aspects of plant metabolism in whole plants including *Nicotiana tabacum* [45], *Panax ginseng* [39] and *Pentalinon andrieuxii* [53]. This tool allows central and secondary plant metabolic pathways to be elucidated under close to unperturbed *in vivo* conditions [42]. The detection of specific distributions of multiply labelled isotopologues in two monoterpenes form ^13^CO_2_-labelled *L. latifolia* confirmed the validity of the experimental approach for the present study.

HMGR in plants is considered the most important enzyme for regulating the MVA pathway; it is regulated at the levels of transcription, post-transcription, translation and post-translation [36]. As expected, overexpression of HMGR enzyme in tobacco, spike lavender, tomato, *Arabidopsis* and ginseng has been reported to increase sterol content [36]. In the case of *Artemisia annua* it was also reported an increase in artemisinin production [54]; in *Salvia miltiorrhiza* total tanshinone production was enhanced [55]; and in *Panax ginseng* triterpene saponin production increased [56]. However, spike lavender is the only plant model were an increase of non-mevalonate metabolites is noted after overexpression of the HMGR enzyme [37].

Results herein do not clearly support that the increased monoterpene production in HMGR overexpressing spike lavender plants are due to a transport of MVA-derived units from the cytoplasm to plastids. It is true that analysis of metabolic flux based on ^13^CO_2_ labelling does not constitute a direct method for the investigation of the cross-talk between both pathways [57]. Nevertheless, the results indicate that the MEP/monoterpene pathway is regulated by a product of the MVA-route at transcriptional, posttranscriptional, translational and/or posttranslational level [13]; therefore, the increased monoterpene production in HMGR overexpressing spike lavender plants could also be explained by feedback MEP pathway gene-regulation in response to HMGR overexpression. As an example, MVA-derived prenylated proteins were shown to regulate the expression of genes in the early steps of monoterpenoid biosynthesis in *Catharanthus roseus* cells [58].

In future experiments, the expression levels of selected enzymes, specifically HMGR before the pulse period, and during/after the chase periods should be monitored in connection with ^13^C-labeling experiments. Secondly, in vitro experiments with [U-^13^C_6_]glucose in combination with inhibitors of the MVA (e.g., mevinoline) and MEP pathways (e.g., fosmidomicine) might help to further clarify the role of a potential crosstalk between the terpene pathways. In any case, it will be important to include other terpenes such as sesquiterpenes, sterols and carotenoids into future studies, in order to finally clarify the distribution of MEP vs. MEV building blocks in the terpenes from *L. latifolia*.

## 4. Materials and Methods

### 4.1. Plant Material

Initial plant material used in this work consisted of *Lavandula latifolia* Med (spike lavender) mature seeds, provided by Intersemillas SA (Valencia, Spain) and in vitro grown transgenic HMGR5 plants overexpressing the HMG1 cDNA, encoding the catalytic domain of 3-hydroxy-3-methylglutaryl CoA reductase (HMGR1S) previously obtained in our group [37]. 

Seeds were sterilized and germinated in vitro as previously described [59]. After germination, seedlings were first transferred to pot-trays with a mixture of peat moss and perlite (7:3) and after 30 days, plantlets were transferred to pots (15 cm) and kept in the greenhouse for 4 months. 

The in vitro HMGR5 and no transgenic wild type (WT) control plants were acclimatized to ex vitro conditions and planted in pots (15 cm) with peat moss and perlite (1:1) as substrate and maintained in a culture chamber for 2 months until used.

### 4.2. ^13^CO_2_ Labelling Experiments

**Figure 10:**
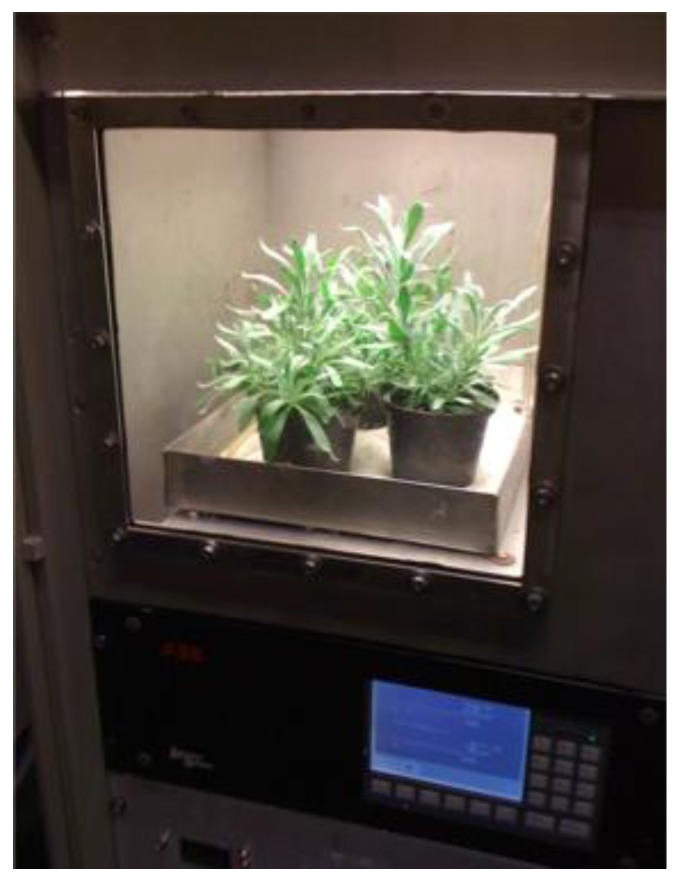
Gas chamber for incubation of plants with ^13^CO_2_.

Labelling experiments were performed with controls and transgenic HMGR5 plants. For ^13^CO_2_ feeding, three or four potted plants were simultaneously placed in a closed gas incubation chamber (Biobox; GWS, Berlin, Germany) at 25 °C and illuminated with white light (Figure 10). Prior to the labelling period (pulse phase), the chamber was flushed with synthetic air containing oxygen and nitrogen until CO_2_ was fully removed. The plants were then fed with synthetic air containing 700 ppm of ^13^CO_2_. During this pulse period, the concentration of ^13^CO_2_ and ^12^CO_2_ was typically detected at a ratio of 9:1. Subsequently, the plants were transferred to the laboratory.

In preliminary experiments, performed with 5-month-old WT plants, pulse and chase periods adequate to produce detectable ^13^C enrichments were determined. To this end, pots were feed for six different pulse periods beginning with 1.0 h up to 8.8 h. Chase periods varied from 0 to 264 h. The time settings for each experiment are listed in Table S1.

Labelling experiments with transgenic HMGR5 plants and their respective controls were performed with 5 h pulse period and chase periods for 96, 168, 240 and 264 h.

Subsequently, leaf essential oils were extracted and the samples were analysed to detect the labelling pattern of camphor and cineole using NMR and/or GC/MS techniques, as described below. Samples indicated with the same lower-case letter belonged to the same plant. 

### 4.3. Essential Oil Extraction from Labelled Material

Depending on the analytical method (GC/MS or ^13^C NMR), two different extraction protocols were employed. 

Samples for GC/MS analysis were extracted as follows: leaf samples (100–200 mg) were introduced into 10 mL glass tubes and 2 mL of chloroform-d (CDCl_3_) was added. After a gentle shake, the tubes were maintained for 15 min at room temperature; subsequently, a spatula of anhydrous sodium sulfate was added and tubes were left for 1 h at room temperature. Finally, 1000 μL of chloroform-extract were placed into a 1.5 mL autosampler vial suitable for GC/MS measurements.

For ^13^C NMR analyses, the plant material (600–1000 mg) was split into 3 different 10 mL glass tubes. Then, 2 mL of chloroform-d was added to the first tube, shacked gently and left at room temperature for 15 min. The chloroform was then transferred into the second tube and the whole process repeated. This procedure was also performed for the third tube. After drying with magnesium sulphate, finally 600 μL of the chloroform extract were placed in a NMR tube for ^1^H and ^13^C analyses.

### 4.4. GC/MS Measurements

The Gas Chromatograph (GC-17A and GC-2010), mass spectrometer (QP-5000 and GCMS-QP 2010 Plus), auto injector (AOC-20i) and software (Class 5000 and GCMS solution) used for these measurements were acquired from Shimadzu (Duisburg, Germany). The column used was a silica capillary column Equity TM-5 (30 m × 0.25 mm × 0.25 μm film thickness) from Supelco Inc. (Bellefonte, PA, USA).

The measurements were performed as follows: the injector and interface temperature were 230 °C and 250 °C respectively. The temperature settings of the oven were: 70 °C for 2 min, then 70–90 °C with 2 °C/min, then 90–130 °C with 5 °C/min and finally 250 °C for 1 min. The pressure program started at 76.1 kPa with a linear velocity of 40.0 cm/s. The flow control was set to linear velocity. The total flow was 16.1 mL/min while the column flow stayed at 1.19 mL/min. The split ratio was 1:10. The detector volts were approx. 0.8 keV (according to the last tuning result of the MS). The solvent cut was adjusted at 4 min and the sampling rate at 0.15 s. The micro scan width was fixed at 0.1 u.

Each sample was analyzed three times in SIM (single ion monitoring) mode. The relative intensities of the standards (camphor and 1,8-cineole) and the samples obtained from GC/MS analysis (peak integration) were processed according to previous publications [60,61,62,63]. This evaluation results in the molar excess of carbon isotopologues of the main components camphor and 1,8-cineole only due to the enrichments from the ^13^C precursor.

GC/MS analysis of the essential oil fraction was performed: (i) to determine the composition of all samples; and (ii) to determine the ^13^C enrichment and isotopologue profiles of the main components in each sample. 

### 4.5. NMR Measurements

NMR analyses were performed as follows: For ^1^H spectra, an Avance I 500 (UltraShield 500 MHz, SEI 500 S2 probe head (5 mm, inverse with Z-gradient), Autosampler B-ACS 60) from Bruker Instruments (Karlsruhe, Germany) was used. The software installed was TopSpin 2.1 (Bruker Instruments, Karlsruhe, Germany). For ^13^C spectra, either an Avance I 500 (Cryomagnet BZH 500 MHz, Autosampler B-ACS 60) or an Advance III 500 system with an UltraShield PLUS 500 MHz magnet and a cryo probe head (5 mm CPQNP, ^1^H/^13^C/^31^P/^19^F/^29^Si (Z-gradient), Autosampler B-ACS 120) both from Bruker Instruments (Karlsruhe, Germany) were used. The software installed was TopSpin 3.0, respectively (Bruker Instruments, Karlsruhe, Germany). The measurements were performed at magnetic fields of 11.75 Tesla. The resonance frequencies of ^1^H and ^13^C were 500.13 MHz and 125.77 MHz, respectively, and the temperature was 300 K. Data analysis was performed with the MestReNova Software (Mestrelab Research, Santiago de Compostela, Spain), TOPSPIN or XWIN NMR. The one-dimensional ^1^H and ^13^C NMR spectra, and the two-dimensional COSY (magnitude mode or phase-sensitive), HSQC, HSQC-DEPTedited, HSQC-TOCSY, NOESY (with 1 s mixing), TOCSY (with 60 ms mixing) and HMBC spectra were measured with standard Bruker parameter sets.

## Figures and Tables

**Table 1:** GC/MS excess data for M+2 and M+3 isotopomeres in 1,8-cineole and camphor extracted with chloroform-d and analyzed by GC/MS from spike lavender leaves at different points of time after the labelling pulse phase with ^13^CO_2_. SD: standard deviation.

				Excess Values (%)			Ratio
	Pulse time (h)	Chase time (h)	Sample	M+2	M+3	M+2/M+3	M+2/(M+2 + M+3)
**Camphor**	4.9 a	95	55	2.72	2.80	0.97	0.49
	5.0 a	168	31	2.78	1.91	1.45	0.59
	5.0 a	264	42	3.65	2.25	1.63	0.62
	5.1 a	16	48	0.29	0.34	0.86	0.46
	5.1 a	16	49	0.44	0.48	0.90	0.47
	5.1 a	40	50	0.54	0.54	1.00	0.50
	5.2 a	192	32	4.86	3.53	1.38	0.58
	5.2 a	240	43	2.10	1.40	1.50	0.60
	8.8 a	240	30	2.40	2.86	0.84	0.46
	-	-	-	-	Mean ± SD	1.17 ± 0.31	0.53 ± 0.07
**Cineole**	4.9 a	95	55	3.81	3.87	0.98	0.50
	5.0 a	168	31	2.52	1.66	1.52	0.60
	5.0 a	264	42	3.43	2.13	1.61	0.62
	5.1 a	16	48	0.44	0.49	0.90	0.47
	5.1 a	16	49	0.55	0.60	0.92	0.48
	5.1 a	40	50	0.58	0.58	1.00	0.50
	5.2 a	192	32	4.87	3.41	1.43	0.59
	5.2 a	240	43	2.13	1.37	1.55	0.61
	8.8 a	240	30	2.61	2.96	0.88	0.47
	-	-	-	-	Mean ± SD	1.20 ± 0.32	0.54 ± 0.07

**Table 2:** NMR Signal assignment for 1,8-cineole.

1,8-cineole Carbon Atom	Signal No.	Chemical Shift (ppm)		-	Multiplicity	Integral	Coupling Constant (Hz)	Correlation					
-	^1^H	^1^H	^13^C (HSQC, HMBC)	-	-	-	-	COSY	COSYph	TOCSY (long range)	HMBC	HSQC	NOESY
3/5	1	2.05	22.80	CH_2_	Multiplet	2	-	2, 3, 4 (w)	2, 3, 4	2, 3, 5	1, 2, 3, 4, 5, 6	1, 3	-
2/6	2	1.69	31.48	CH_2_	Multiplet	2 (2.4)	-	1, 3	1, 3	1, 3, 4	1, 2, 3, 4, 6 (strong)	2, 3	-
-	3	1.52	22.80 31.48	CH_2_	Multiplet	4	-	1, 2	1, 2	1, 2, 4	-	-	-
4	4	1.43	32.91	CH	Multiplet	1	-	1 (weak)	1	1, 2, 3	1, 2, 3, 5 (s)	4	-
9/10	5	1.26	28.87	CH_3_	Singulet	6	-	-	-	-	4, 5 (strong)	5	-
7	6	1.07	27.55	CH_3_	Singulet	3	-	-	-	-	2 (weak), 3 (weak)	6	-
1	-	-	69.92	-	-	-	-	-	-	-	1, 2, 3 (weak), 6 (strong)	-	-
8	-	-	73.76	-	-	-	-	-	-	-	1, 2 (weak), 3, 5 (strong), 6 (weak)	-	-

**Table 3:** NMR Signal assignment for camphor.

Camphor Carbon Atom	Signal No.	Chemical Shift (ppm)		-	Multiplicity	Integral	Coupling Constant (HZ)	Correlation					
-	^1^H	^1^H	^13^C (HSQC, HMBC)	-	-	-	-	COSY	COSYph	TOCSY (long range)	HMBC	HSQC	NOESY
3	1	2.36	43.32	CH_2_	ddd	1	18.0 4.5 3.3	2, 4	2, 4, 7	2, 3	1, 3, 4, 5+6+7 (weak), 8, 10	1, 2, 4	-
4	2	2.10	43.04	CH	Triplet	1	4.5	1	1	1, 3, 4, 5, 6, 7	1, 3, 4, 5+6+7 (weak), 8, 10	1, 2, 4	-
5	3	1.96	27.04	CH_2_	Multiplet	1	-	5, 7	5, 7	1, 2, 5, 6, 7	1, 2 (weak), 4, 5, 6	3, 7	-
3	4	1.85	43.32	CH_2_	Doublet	1	18.2	1	1	2	1, 3, 4, 5+6+7 (weak), 8, 10	1, 2, 4	-
6	5	1.69	29.91	CH_2_	dd	1	13.1 4.1	3, 6	3, 6	3, 6, 7	2, 3, 7, 9	5, 6	-
6	6	1.41	29.91	CH_2_	Multiplet	1	-	5, 7	5, 7	2, 3, 5, 7	2, 3, 7, 9	5, 6	-
5	7	1.35	27.04	CH_2_	Multiplet	1	-	3, 6	3, 6	2, 3, 5, 6	1, 2 (weak), 4, 5, 6	3, 7	-
9	8	0.97	19.15	CH_3_	Singulet	3	-	-	-	-	10	8	5, 3
10	9	0.92	9.27	CH_3_	Singulet	3	-	-	-	-	5	9	5
8	10	0.85	19.80	CH_3_	Singulet	3	-	-	-	-	2, 4, 8	10	1
2	-	-	220.06	CH_2_	ddd	1	-	-	-	-	1, 2, 4, 5, 6, 9	-	-
1	-	-	57.76	CH	Triplet	1	-	-	-	-	2, 3+4 (weak), 5, 6, 7, 8, 9, 10	-	-
7	-	-	46.84	CH_2_	Multiplet	1	-	-	-	-	4, 6, 7, 8, 9, 10	-	-

**Table 4:** NMR analysis of camphor and 1,8-cineole derived from chloroform-d extraction of spike lavender leaves pulse-labelled with ^13^CO_2_ for 5.2 h; chase time 192 h.

Position Carbon Atoms	Chemical Shift [ppm]	^13^C Coupling	^13^C-^13^C Coupling Constant [Hz]
Camphor			
10	9.27	to C1	41.1
9	19.15	to C7	37.9
8	19.8	to C5	2.5
5	27.04	to C4 to C8	32.2 2.5
6	29.91	to C2	2.3
4	43.04	to C5	32.2
3	43.32	to C2	34.3
7	46.84	to C9	37.9
1	57.76	to C10	41.1
2	220.06	to C6	2.3
1,8-cineole			
3/5	22.8	C3–C4	32.9
7	27.55	to C1	43.3
9/10	28.87	C10–C8 C9–C3/4	41.0 2.2
2/6	31.48	-	?
4	32.91	to C3	32.9
1	69.93	to C7	43.3
8	73.76	to C10	41

**Table 5:** Main components of spike lavender leaf essential oil from lines HMGR5 and WT extracted with chloroform-d and determined by GC/MS. The percentage is referred to the area of the 15 main peaks of each sample. Rt: retention time; SD: standard deviation; h: hours.

Sample and Chase Period	α-Pinene rt: 5.9	β-Pinene rt: 7.2	Cineole rt: 9.2	Limonene rt: 9.0	Camphor rt: 14.3
HMGR5 plants	-	-	-	-	-
96 h	8.15	6.77	53.78	2.09	16.83
96 h	2.79	1.77	53.87	0.84	33.01
96 h	5.33	3.42	49.91	1.79	30.49
168 h	3.63	2.14	37.56	1.02	28.88
168 h	2.37	1.58	52.94	0.65	31.96
240 h	4.87	3.33	48.44	1.73	27.94
240 h	5.02	2.98	50.99	1.27	30.42
240 h	4.22	2.64	52.82	1.39	33.47
Mean ± SD	4.55 ± 1.80	3.08 ± 1.64	50.04 ± 5.40	1.35 ± 0.50	29.13 ± 5.32
WT plants	-	-	-	-	-
96 h	4.8	3.05	49.83	1.54	29.23
264 h	5.89	4.81	44.54	3.24	19.43
264 h	5.54	4.5	42.8	3.84	12.13
264 h	7.08	4.8	32.6	3.84	29.27
Mean ± SD	5.83 ± 0.95	4.29 ± 0.84	42.44 ± 7.21	3.12 ± 1.09	22.52 ± 8.33
Sample and chase period	α-pinene rt: 5.9	β-pinene rt: 7.2	Cineole rt: 9.2	Limonene rt: 9.0	Camphor rt: 14.3

**Table 6:** GC/MS excess data for M+2 and M+3 isotopomeres in camphor extracted with chloroform-d and analyzed by GC/MS from spike lavender leaves from lines HMGR5 and WT at different points of time after the labelling pulse phase with ^13^CO_2_. h: hours.

		Excess Values (%)		Ratio	
-	-	M+2	M+3	M+2/M+3	M+2
					(M+2) + (M+3)
Camphor	HMGR5	-	-	-	-
-	96 h	0.30	0.17	1.76	0.64
-	96 h	0.05	0.02	2.50	0.71
-	96 h	0.20	0.11	1.82	0.65
-	168 h	0.05	0.02	2.50	0.71
-	168 h	0.03	0.02	1.50	0.60
-	240 h	0.32	0.17	1.88	0.65
-	240 h	0.29	0.13	2.23	0.69
-	240 h	0.15	0.11	1.36	0.58
-	WT	-	-	-	-
-	96 h	0.30	0.17	1.76	0.64
-	96 h	0.05	0.02	2.50	0.71
-	96 h	0.20	0.11	1.82	0.65
-	168 h	0.05	0.02	2.50	0.71
-	168 h	0.03	0.02	1.50	0.60

**Table 7:** GC/MS excess data for M+2 and M+3 isotopomeres in 1,8-cineole extracted with chloroform-d and analyzed by GC/MS from spike lavender leaves from lines HMGR5 and WT at different points of time after the labelling pulse phase with ^13^CO_2_. h: hours.

		Excess Values (%)		Ratio	
-	-	M+2	M+3	M+2/M+3	M+2
					(M+2) + (M+3)
1,8-cineole	HMGR5	-	-	-	-
-	96 h	0.27	0.16	1.69	0.63
-	96 h	0.22	0.12	1.83	0.65
-	168 h	0.07	0.04	1.75	0.64
-	240 h	0.21	0.11	1.91	0.66
-	240 h	0.32	0.14	1.79	0.70
-	240 h	0.17	0.12	1.42	0.59
-	WT	-	-	-	-
-	96 h	0.05	0.03	1.67	0.63
-	264 h	0.53	0.27	1.96	0.66
-	264 h	0.15	0.09	1.67	0.63
	264 h	0.87	0.48	1.81	0.64

## Supplementary Materials

The following are available online at www.mdpi.com/2218-1989/7/4/65/s1, Table S1: Experimental overview, preliminary experiments, Table S2: Absolute ^13^C enrichments in 1,8-cineole for each carbon atom as calculated from ^13^C NMR and GC/MS, Table S3: Main components of spike lavender leaf essential oil, including coumarine, from lines HMGR5 and WT extracted with chloroform-d and determined by GC/MS. Table S4: Experimental overview of WT and HMGR5 lines. Pulse period of 5 h for all samples, Figure S1: Isotopologue excess values and distribution of isotopomers of cineole for 51 ^13^CO_2_ feeding experiments, Figure S2: Isotopologue excess values and distribution of isotopomers of camphor in 28 ^13^CO_2_ feeding experiments.
