# Supplementary material for: Dynamics of Monoterpene Formation in Spike Lavender Plants
Source: Metabolites. 2017 Dec 19;7(4):65. doi: 10.3390/metabo7040065 (PMC5746745; doi:10.3390/metabo7040065)
Supplement: Supplementary file 1 [file metabolites-07-00065-s001.zip › Supplemental/Supplemental Figures.pdf]

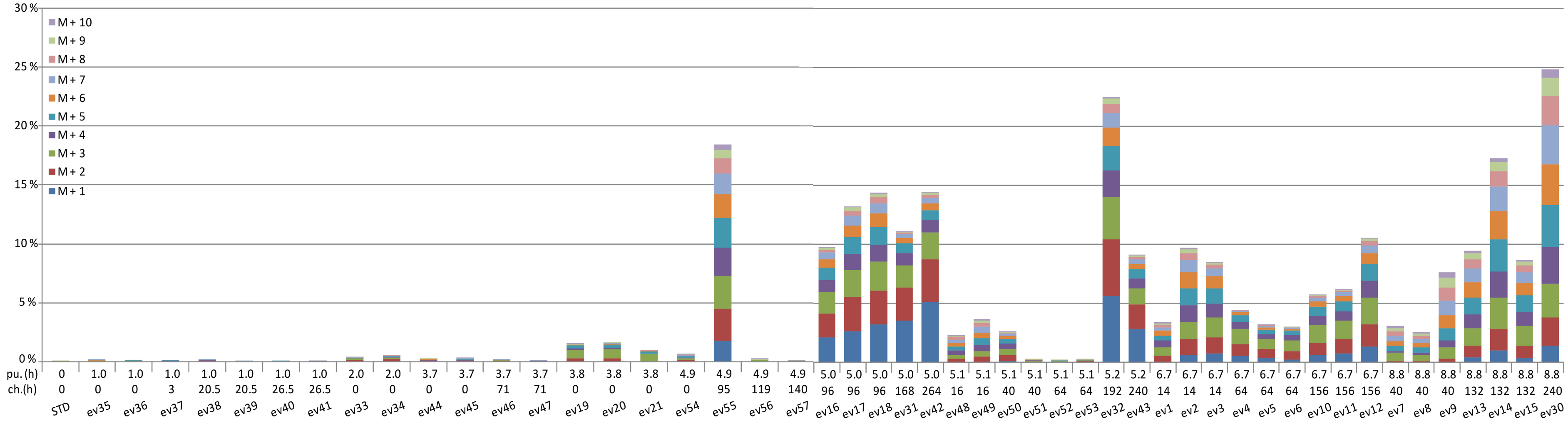

Figure S1: Isotopologue excess values and distribution of isotopomers of camphor for 51 $^{13}CO_2$ feeding experiments. All experiments are sort according to their chase time in ascending order. pu. (h): pulse time in hours. ch. (h): chase time in hours. STD: pure camphor sample

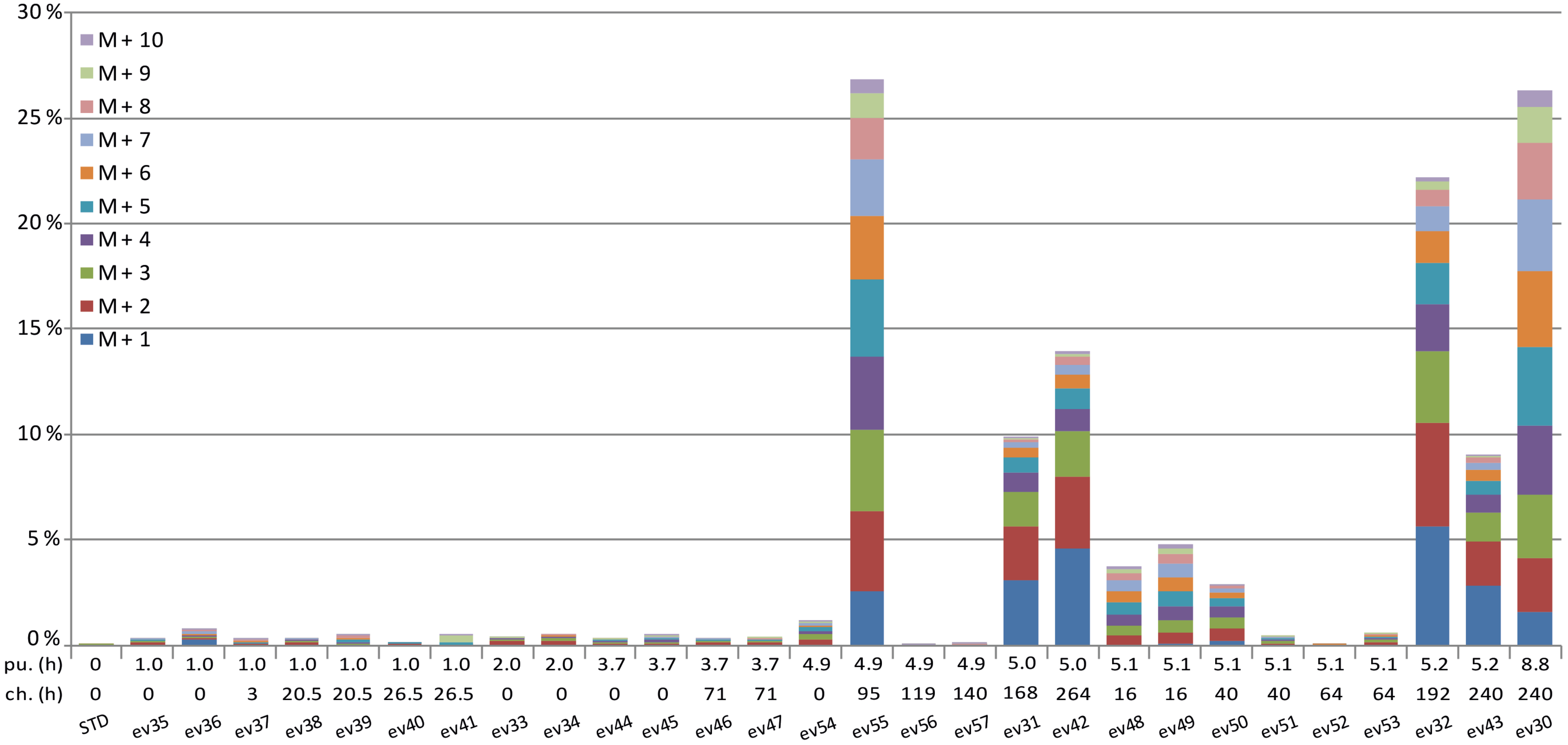

Figure S2: Isotopologue excess values and distribution of isotopomers of cineole in 28 $^{13}CO_2$ feeding experiments. All experiments are sort according to their chase time in ascending order. pu. (h): pulse time in hours. ch. (h): chase time in hours. STD: pure cineole sample
