# Supplementary material for: Dynamics of Monoterpene Formation in Spike Lavender Plants
Source: Metabolites. 2017 Dec 19;7(4):65. doi: 10.3390/metabo7040065 (PMC5746745; doi:10.3390/metabo7040065)
Supplement: Supplementary file 1 [file metabolites-07-00065-s001.zip › Supplemental/Supplemental Tables.pdf]

Table S1. Experimental overview, preliminary experiments.

					Camphor			Cineol		
Sample	Pulse period (h)	Chase period (h)	$^{13}CO_2$ (ml)	mg material	Excess ^{13}C abundance (%)	^{13}C isotopologues excess (%)	Percentage M+1- M+3	Excess ^{13}C abundance (%)	^{13}C isotopologues excess (%)	Percentage M+1- M+3
1	6.73	14	200	128.8	1.58	n.d.	n.d.	n.d.	n.d.	n.d.
2	6.73	14	200	126.9	4.47	n.d.	n.d.	n.d.	n.d.	n.d.
3	6.73	14	200	130.3	3.47	n.d.	n.d.	n.d.	n.d.	n.d.
4	6.73	64	200	124	1.40	n.d.	n.d.	n.d.	n.d.	n.d.
5	6.73	64	200	124.8	1.07	n.d.	n.d.	n.d.	n.d.	n.d.
6	6.73	64	200	121.9	1.04	n.d.	n.d.	n.d.	n.d.	n.d.
7	8.84	40	300	131.8	1.81	n.d.	n.d.	n.d.	n.d.	n.d.
8	8.84	40	300	121.7	1.42	n.d.	n.d.	n.d.	n.d.	n.d.
9	8.84	40	300	156.4	4.73	n.d.	n.d.	n.d.	n.d.	n.d.
10	6.73	156	200	173.1	2.13	n.d.	n.d.	n.d.	n.d.	n.d.
11	6.73	156	200	159.7	2.23	n.d.	n.d.	n.d.	n.d.	n.d.
12	6.73	156	200	180.8	4.03	n.d.	n.d.	n.d.	n.d.	n.d.
13	8.84	132	300	184.5	4.74	n.d.	n.d.	n.d.	n.d.	n.d.
14	8.84	132	300	139.4	8.49	n.d.	n.d.	n.d.	n.d.	n.d.
15	8.84	132	300	152.6	4.07	n.d.	n.d.	n.d.	n.d.	n.d.
16	5.00	96	n.d.	121.3	3.33	n.d.	n.d.	n.d.	n.d.	n.d.
17	5.00	96	n.d.	138.3	4.67	n.d.	n.d.	n.d.	n.d.	n.d.
18	5.00	96	n.d.	160.8	5.01	n.d.	n.d.	n.d.	n.d.	n.d.
19	3.79	0	n.d.	142.6	0.53	n.d.	n.d.	n.d.	n.d.	n.d.
20	3.79	0	n.d.	152.3	0.58	n.d.	n.d.	n.d.	n.d.	n.d.
21	3.79	0	n.d.	132	0.38	n.d.	n.d.	n.d.	n.d.	n.d.
30	8.84	240	300	500	13.02	24.87	26.69	13.73	26.29	27.06
31	5.00	168	225	400	3.00	11.08	74.22	2.69	9.86	73.74
32	5.21	192	161	400	7.54	22.54	61.98	7.32	22.17	62.84
33	2.00	0	n.d.	400	0.11	0.33	n.d.	0.10	0.35	n.d.

n.d.: not determined

Table S1. Experimental overview, preliminary experiments.

					Camphor			Cineol		
Sample	Pulse period (h)	Chase period (h)	$^{13}CO_2$ (ml)	mg material	Excess ^{13}C abundance (%)	^{13}C isotopologues excess (%)	Percentage M+1- M+3	Excess ^{13}C abundance (%)	^{13}C isotopologues excess (%)	Percentage M+1- M+3
34	2.00	0	n.d.	400	0.16	0.49	n.d.	0.15	0.46	n.d.
35	1.00	0	n.d.	400	0.04	0.11	n.d.	0.09	0.28	n.d.
36	1.00	0	n.d.	400	0.02	0.08	n.d.	0.28	0.73	n.d.
37	1.00	3	n.d.	380	0.02	0.05	n.d.	0.24	0.37	n.d.
38	1.00	20.5	n.d.	400	0.05	0.14	n.d.	0.09	0.30	n.d.
39	1.00	20.5	n.d.	400	0.01	0.02	n.d.	0.28	0.50	n.d.
40	1.00	26.5	n.d.	400	0.01	0.04	n.d.	0.03	0.09	n.d.
41	1	26.5	n.d.	400	0.01	0.03	n.d.	0.39	0.50	n.d.
42	5	264	225	403	3.86	14.42	76.06	3.95	13.83	72.77
43	5.21	240	161	403	2.66	9.05	29.32	2.63	9.00	69.84
44	3.74	0	127	397	0.08	0.20	n.d.	0.11	0.30	n.d.
45	3.74	0	127	400	0.13	0.27	n.d.	0.21	0.45	n.d.
46	3.74	71	127	393	0.05	0.16	n.d.	0.08	0.26	n.d.
47	3.74	71	127	393	0.03	0.08	n.d.	0.11	0.31	n.d.
48	5.1	16	167.5	410	1.20	2.31	26.95	1.97	3.72	25.01
49	5.1	16	167.5	417	1.97	3.68	25.05	2.55	4.78	25.05
50	5.1	40	167.5	410	1.04	2.53	44.99	1.14	2.86	47.04
51	5.1	40	167.5	417	0.09	0.20	n.d.	0.17	0.40	n.d.
52	5.1	64	167.5	400	0.05	0.12	n.d.	0.02	0.05	n.d.
53	5.1	64	167.5	400	0.09	0.23	n.d.	0.20	0.51	n.d.
54	4.92	0	162.5	413	0.24	0.57	n.d.	0.47	1.12	n.d.
55	4.92	95	162.5	413	8.33	18.41	39.54	12.35	26.83	38.1
55-2	4.92	95	162.5	1090	n.d.	n.d.	n.d.	n.d.	n.d.	n.d.
56	4.92	119	162.5	403	0.07	0.17	n.d.	0.01	0.03	n.d.
57	4.92	139.5	162.5	403	0.05	0.10	n.d.	0.04	0.06	n.d.
58	4.92	408	162.5	393	n.d.	n.d.	n.d.	n.d.	n.d.	n.d.
59	4.92	408	162.5	1100	n.d.	n.d.	n.d.	n.d.	n.d.	n.d.

n.d.: not determined

Table S2: Absolute ^{13}C enrichments in 1,8-cineole for each carbon atom as calculated from ^{13}C NMR and GC/MS

1,8-cineole carbon atom	^{13}C Shift [ppm]	Integral unlabeled Cineol	Integral labeled cineol from ev32	rel. ^{13}C [%]	abs. ^{13}C [%]
1	69.92	1052	3207	3.05	8.87
2/6	31.48	4297	13450	3.13	8.63
3/5	22.8	4238	13504	3.19	8.48
4	32.91	2011	6453	3.21	8.42
7	27.55	2111	7153	3.39	7.98
8	73.76	1000	3303	3.30	8.18
9/10	28.87	4289	13963	3.26	8.30

Table S3: Main components of spike lavender leaf essential oil, including coumarine, from lines HMGR5 and WT extracted with chloroform-d and determined by GC/MS. The percentage is referred to the area of the 15 main peaks of each sample. Rt: retention time SD: standard deviation h: hours

Sample and chase period	α-pinene rt: 5.9	β-pinene rt:7.2	cineole rt: 9.2	limonene rt: 9.0	camphor rt: 14.1	coumarine rt:23.4	Total
HMGR5 plants							
96 h	8.15	6.77	53.78	2.09	16.83	4.68	92,32
96 h	2.79	1.77	53.87	0.84	33.01	2.93	95,21
96 h	5.33	3.42	49.91	1.79	30.49	2.97	93,90
168 h	3.63	2.14	37.56	1.02	28.88	20.37	93,61
168 h	2.37	1.58	52.94	0.65	31.96	4.58	94,08
240 h	4.87	3.33	48.44	1.73	27.94	8.21	94,52
240 h	5.02	2.98	50.99	1.27	30.42	4.16	94,83
240 h	4.22	2.64	52.82	1.39	33.47	0,00	94,54
Mean ± SD	4.55 ± 1.80	3.08 ± 1.64	50.04 ± 5.40	1.35 ± 0.50	29.13 ± 5.32	5.99±6.25	94.13±0.89
WT plants							
96 h	4.8	3.05	49.83	1.54	29.23	1.71	90.15
264 h	5.89	4.81	44.54	3.24	19.43	12.22	90.13
264 h	5.54	4.5	42.8	3.84	12.13	12.51	81.32
264 h	7.08	4.8	32.6	3.84	29.27	13.93	91.52
Mean ± SD	5.83 ± 0.95	4.29 ± 0.84	42.44 ± 7.21	3.12 ± 1.09	22.52 ± 8.33	10.09±5.64	88.28 ± 4.68

Table S4 Experimental overview of WT and HMGR5 lines. Pulse period of 5 hours for all samples

Line	Chase period (hours)	Camphor excess ^{13}C abundance percentage	Cineol excess ^{13}C abundance percentage
HMGR5	96	0.20	0.18
HMGR5	96	0.02	0.02
HMGR5	96	0.11	0.12
HMGR5	168	0.02	0.04
HMGR5	168	0.02	0.01
HMGR5	240	0.30	0.19
HMGR5	240	0.14	0.15
HMGR5	240	0.15	0.16
WT	96	0.03	0.04
WT	264	0.44	0.37
WT	264	0.15	0.10
WT	264	0.83	0.93
